# Expression of matrix metalloproteinase-1 (MMP-1) in Wistar rat's intervertebral disc after experimentally induced scoliotic deformity

**DOI:** 10.1186/1748-7161-6-9

**Published:** 2011-05-09

**Authors:** Theodoros B Grivas, Elias S Vasiliadis, Angelos Kaspiris, Lubna Khaldi, Dimitris Kletsas

**Affiliations:** 1 Orthopaedic Department, "Tzanio" General Hospital, Piraeus, Greece; 2 Orthopaedic Department, "Thriasio" General Hospital, Athens, Greece; 3 Department of Pathology, "A. Fleming" General Hospital, Athens, Greece; 4 Laboratory of Cell Proliferation & Ageing, Institute of Biology NCSR "Demokritos", Athens, Greece

## Abstract

**Introduction:**

A scoliotic deformity on intervertebral discs may accelerate degeneration at a molecular level with the production of metalloproteinases (MMPs). In the present experimental study we evaluated the presence of MMP-1 immunohistochemically after application of asymmetric forces in a rat intervertebral disc and the impact of the degree of the deformity on MMP-1 expression.

**Material-Method:**

Thirty female Wistar rats (aged 2 months old, weighted 200 ± 10 grams) were used. All animals were age, weight and height matched. A mini Ilizarov external fixator was applied at the base of a rat tail under anaesthesia in order to create a scoliotic deformity of the intervertebral disc between the 9^th ^and 10^th ^vertebrae. Rats were divided into three groups according to the degree of the deformity. In group I, the deformity was 10°, in group II 30° and in group III 50°. The rats were killed 35 days after surgery. The discs were removed along with the neighbouring vertebral bodies, prepared histologically and stained immunohistochemically. Immunopositivity of disc's cells for MMP-1 was determined using a semi-quantitative scored system.

**Results:**

MMP-1 immunopositivity was detected in disc cells of annulus fibrosus of all intervertebral disc specimens examined. The percentage of MMP-1 positive disc cells in annulus fibrosus in group I, II and III were 20%, 43% and 75%, respectively. MMP-1 positivity was significantly correlated with the degree of the deformity (p < 0,001). An increase of chondrocyte-like disc cells was observed in the outer annulus fibrosus and at the margin of the intervertebral disc adjacent to the vertebral end plates. The difference in the proportion of MMP-1 positive disc cells between the convex and the concave side was statistically not significant in group I (p = 0,6), in group II this difference was statistically significant (p < 0,01). In group III the concave side showed a remarkable reduction in the number of disc's cells and a severe degeneration of matrix microstructure.

**Conclusion:**

The present study showed that an experimentally induced scoliotic deformity on a rat tail intervertebral disc results in over-expression of MMP-1, which is dependent on the degree of the deformity and follows a dissimilar distribution between the convex and the concave side.

## Introduction

The matrix of the intervertebral disc is an avascular tissue which consists of a collagen network and a proteoglycan gel which provides the disc with the properties necessary to fulfill its function of withstanding compressive and torsional forces. The matrix of the central nucleus pulposus is rich in proteoglycans, whereas the anulus fibrosus is predominantly collagenous [1-3]. The collagen is type I and type II and is distributed radially in opposing concentration gradients, with type I collagen mainly comprising the fiber bundles of the anulus fibrosus, whereas type II collagen is the principal component of the random fibrillar network of the nucleus pulposus [4]. In addition, the minor collagen types III, V, VI, IX, and XI are present, at varying degrees, across the disc [5].

Mechanical forces on intervertebral discs influence the metabolic behavior of both the disc cells and the extracellular matrix [6-9]. Asymmetric forces on the intervertebral disc in vivo is expected to result in deformation patterns for fibroblast-like cells and for fiber bundles of type I collagen of the highly oriented anulus fibrosus. The response to mechanical stimuli depends on the loading type, magnitude, duration, and anatomic zone of cell origin [7,9,10]. This response may accelerate degeneration at a molecular level with the production of metalloproteinases (MMPs). Structural changes at a microscopic level in the collagenous network will have important consequences for the flexibility and mechanical properties of the disc, because these properties are dependent on the structure of the collagen fibrils [11].

The MMPs are a family of peptidase enzymes responsible for the degradation of extracellular matrix components. The fibrillar collagens are broken down initially by the collagenases which belong to the extended family of matrix metalloproteinases [12]. Especially, the interstitial collagenase, MMP-1, cleave the fibrillar collagens types I, II, and III at a single site in the molecule.

Various investigators have studied aspects of the biochemistry of the intervertebral disc in idiopathic scoliosis. There are differences in the distribution of type I and type II collagen between the concave and convex side of the curve [13] and a reduction in the collagen content on the concave side of the deformity [14]. In addition, differences in the composition and distribution of various glycosaminoglycans in scoliotic discs have been demonstrated [15,16].

Although the association between adult disc degeneration and MMPs has previously been reported [17], the expression of MMP-1 in scoliotic intervertebral discs in a growing spine has not been studied yet. In the present experimental study we evaluated immunohistochemically the expression of MMP-1 after application of asymmetric forces to a rat intervertebral disc. We also investigated the degree of deformity and its impact on the production of MMP-1, as well as the possible aetiopathogenic role of this mechanism in disc degeneration and progression of the scoliotic curve.

## Material and methods

### Animal model

Thirty female Wistar rats were selected after controlled breeding. Animals were included in the study before their adulthood in order to take into account the impact of growth for the study of intervertebral disc changes and were categorized into three groups (of 10 rats each) according to the degree of the deformity. In group I, the deformity which was applied on the intervertebral disc was 10°, in group II 30° and in group III 50°. All animals were age, weight and height matched. They were two months old and weighted 200 ± 10 grams. Laboratory conditions included stable humidity, temperature of 21°C and artificial daylight for 12 hours. Criteria for an animal to be excluded from the study were a) deviation of weight or height beyond two standard deviations because in animals with such differences there are problems in evaluation of tissue strength and b) major health problems which could potentially affect bone metabolism. All animal procedures were reviewed and approved by the Veterinary Ethics Committee.

### Experimental procedure

**Figure 1:**
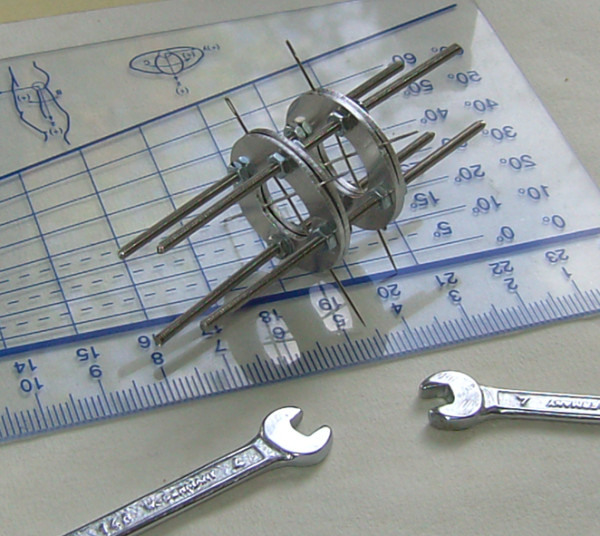
**The loading apparatus; an Ilizarov type mini external fixator**.

The experiment was performed with the use of a loading apparatus which has been previously described [18] and was modified accordingly for the needs of the present study (Figure 1). The loading apparatus was an Ilizarov type mini external fixator which was applied at the base of a rat tail under anaesthesia. Anesthesia was performed by peritoneal injection of 80 mg/kg ketamine. The intact intervertebral discs outside of the fixator were used as controls.

The Ilizarov mini external fixator consists of two double rings and four threaded rods. Each ring was made of aluminum in order to be extremely light, had a diameter of 25 mm and weighted 1 gr. It had a central hole with a diameter of 13 mm and four smaller holes 3 mm in diameter, where the 2 mm connecting rods were adjusted and secured with M2 nuts (Figure 1).

The rat tail was prepared after anesthesia and the 9^th ^and 10^th ^vertebrae at its base were palpated and marked. Then two sterile thin stainless steel wires (0,8 mm in diameter) were inserted through each of the 9^th ^and 10^th ^vertebral body at a 90° angle with the use of a mini power drill. Each pair of wires was placed between the two single rings of the double ring. At the end of this procedure each of the two vertebral bodies (9^th ^and 10^th^) were fixed to a double ring with the two transosseous wires. The two double rings which were pre-connected with the four connecting rods were tightened with the nuts at a pre-determined angle according to the protocol of the study and an accurate angular deformity of the intervertebral disc between the 9^th ^and 10^th ^vertebrae of the rat tail was achieved.

**Figure 2:**
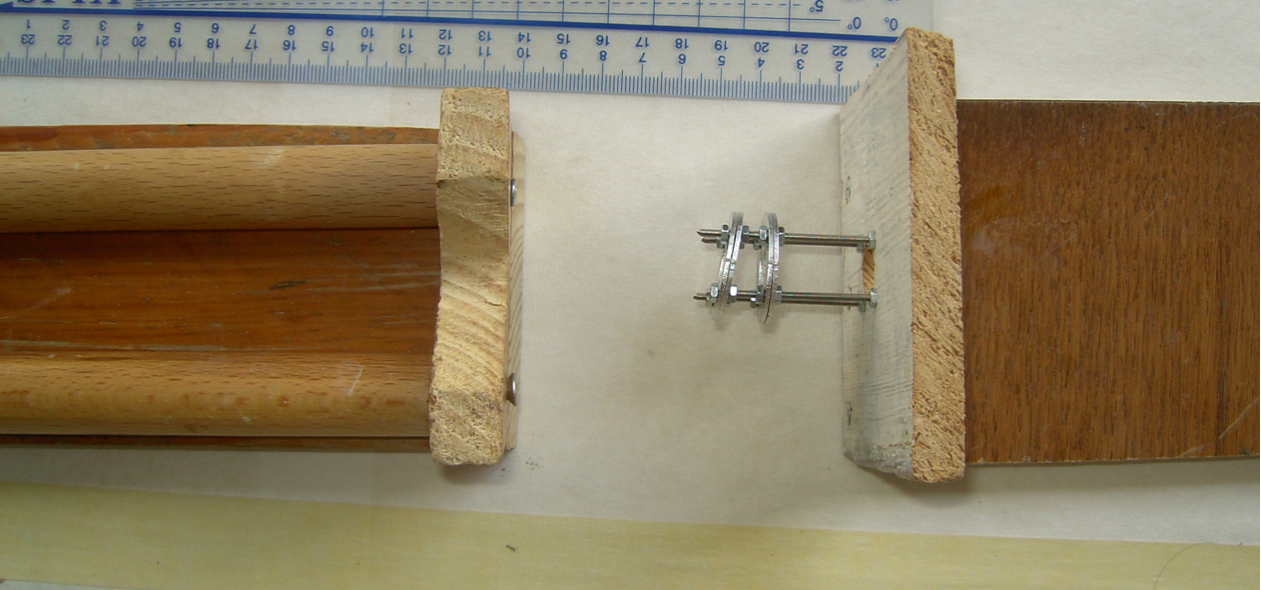
**The specific bed used for the application of the loading apparatus**. The two double rings were pre-fastened to the bed and the connecting rods were pre-positioned at a pre-determined angle.

**Figure 3:**
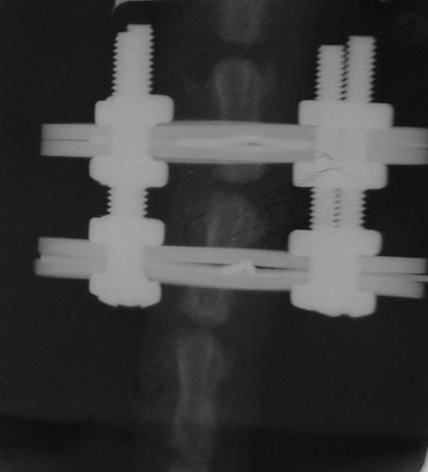
**An x-ray showing the accurate position of the wires through the vertebral bodies and the angular deformity of the intervertebral disc**.

In order to ensure accurate placement of the wires through the vertebral bodies of the 9^th ^and 10^th ^vertebrae and fixation onto the double rings, a specific bed was designed and constructed (Figure 2). The two double rings were pre-fastened to the bed and the connecting rods were pre-positioned. The two double rings were placed at a pre-determined angle of 10°, 30°, or 50°, depending on which group the rat belonged. Initially the anesthetized rat was placed supine in a way that the axis of the spine runs flat along the bed and two side supports ensured that the tail was always seated in the centre of the double rings. Then the two pairs of wires were drilled between the two rings of each double ring and tightened with the nuts. Finally the two double rings were forced to become parallel and the intermediate intervertebral disc sustained an angular deformity. Accurate placement of the transosseous wires was checked by intra-operative x-rays (Figure 3).

### Sample harvest and preparation

After rats' sacrificed on 35^th ^day, the tails were surgically prepared, the skin was removed and specimens were dipped in 10% buffer formalin.

### Histological & immunohistochemical evaluation

The specimens were fixed in 10% buffer formalin for 24-36 h, then decalcified in neutral EDTA for 6 weeks in room temperature, and embedded in paraffin blocks. Histological sections 3 μm thick were obtained and stained with Hematoxylin/Eosin.

For immunohistochemical staining the sections were deparaffinized in xylene and degraded alcohols, immersed in distilled water, blockage of endogenous peroxidase were achieved with 3% H_2_O_2 _for 30 minutes in a dark chamber at room temperature. Sections were then washed in distilled water and 3 times with TBS, incubated for 1 hour at room temperature with anti-MMP1/8 (H-300) (Santa Cruz sc-30069) diluted 1:50 in antibody diluent (DAKO REAL S2022), incubated for 45 minutes at room temperature with peroxidase-labeled anti-mouse/rabbit IgG (En-vision Kit, DAKO Detection System, Peroxidase/DAB+, Rabbit/Mouse K5007), washed 3 times with TBS, and stained for 10 minutes in a dark chamber at room temperature with 3-amino-9-ethylcarbazole/H_2_O_2_, washed in distilled and counterstained with hematoxylin.

### Scoring of Intervertebral Disc Specimens for Immunopositivity

Each disc specimen was scored semiquantitatively for immunopositivity by determining the proportion of disc cells that were positive for MMP-1 [19]. Scoring was determined by calculating the number of positive disc cells over a minimum of 10 random high power fields of view using the Χ20 objective in both the convex and the concave side of the disc. Only disc cells that were clearly MMP-1 positive were scored. Microscope fields containing no immunopositive cells were scored as 0; those containing 1-10 positive cells were scored as 1; those containing 11-20 positive cells were scored as 2 and those containing >20 positive cells were scored as 3. The score of 0, 1, 2 and 3 was determined for each microscope field. Then the mean score for the overall disc and for the convex and concave side were used for statistical analysis.

### Statistical Analysis

The nonparametric Mann-Whitney U test was used to determine significant differences between MMP-1 positivity, in each group of deformity. Mann-Whitney U test was used for assessing whether the independent scores of the microscope fields between the three groups, or between the convex and concave side were statistically significant. Statistically significance was designated at the p < 0.05 level.

## Results

**Figure 4:**
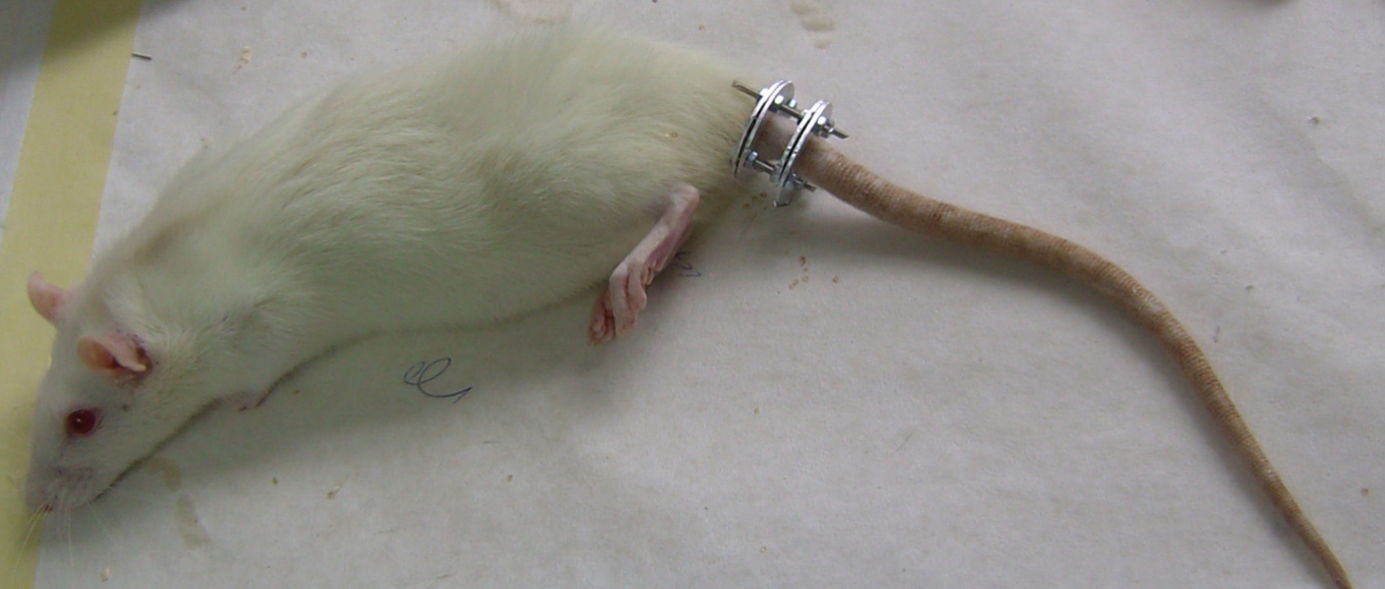
**An anesthetized rat with the loading apparatus fixed at the base of its tail**. The fixator was well tolerated by all the animals for the whole period it was applied.

In the present study two animals died immediately after the injection of ketamine and were substituted. One additional animal was excluded from the study because of necrosis of the tail distal to the fixator and was replaced. Ilizarov mini external fixator was well tolerated by all the animals for the whole period it was applied (Figure 4).

### Immunohistochemistry

**Figure 5:**
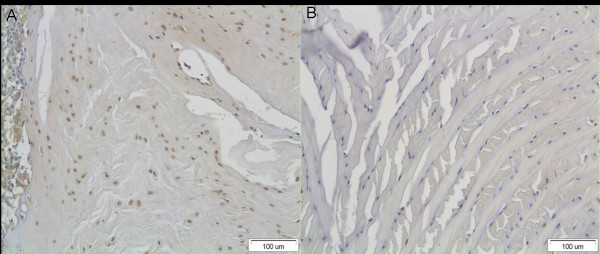
**A) An increase of the expression of the MMP-1 in the examined disc specimen after the application of asymmetrical load through the external fixator**. B) The intact control disc which was outside of the fixator and sustained no forces.

**Figure 6:**
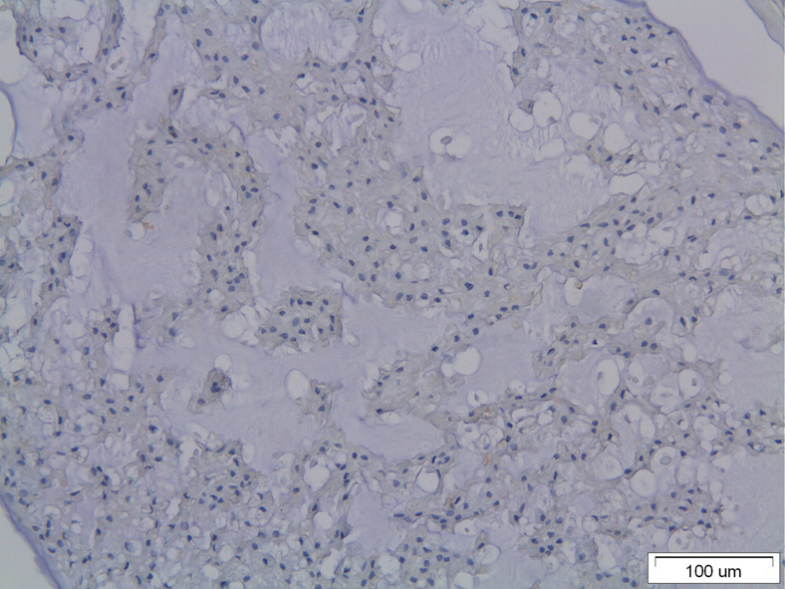
**The nucleus pulposus of the examined disc specimen**. Note the absence of immunopositive disc cells.

MMP-1 immunopositivity was detected in disc cells of all intervertebral disc specimens examined. An increase of the expression of the MMP-1 was found in all groups compared with the controls (Figure 5). Interestingly, MMP-1 was not detected at the nucleus pulposus (Figure 6).

**Figure 7:**
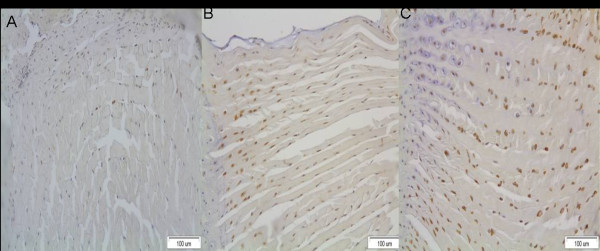
**A positive correlation between the degree of the deformity and the quantity of MMP-1 expression**. MMP-1 positive disc cells in annulus fibrosus in group I, II and III were 20%, 43% and 75%, respectively A) disc from group I, B) disc from group II, C) disc from group III.

There was a positive correlation between the degree of the deformity and the number of immunereactive for MMP-1 cells (Figure 7). Thus, the number of those cells increased as the degree of the deformity progressed. The frequency of MMP-1 positive cells in annulus fibrosus in group I, II and III were 20%, 43% and 75%, respectively. MMP-1 positivity was significantly correlated with the degree of the deformity (p < 0,001) (Table 1).

**Table 1:** Shows the proportion of disc cells positive for MMP 1 among the three groups and the mean score of each group.

	Group I	Group II	Group II	p value
**Proportion of MMP 1 positive disc cells**	20%	43%	75%	

**Score**	**1,2**	**2,5**	**3**	**<0,001**

The score, which was used for statistical analysis, was determined by calculating the number of positive disc cells over a minimum of 10 random high power fields of view using the Χ20 objective. 0: no immunopositive cells; 1: 1-10 positive cells; 2: 11-20 positive cells and 3: >20 positive cells.

**Figure 8:**
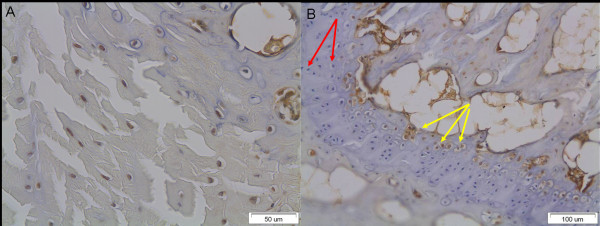
**Increased number of chondrocyte-like disc cells at A) the transition zone and B) the margin of the intervertebral disc adjacent to the vertebral end plates**. Note the difference between the normal chondrocytes (red arrows) and other chondrocyte-like cells, which were immunopositive for MMP-1 (yellow arrows).

MMP-1 immunopositivity was most marked in the outer anulus fibrosus. At the transition zone of all groups, clusters of chondrocyte-like disc cells emerged and some were MMP-1 positive (Figure 8). A similar increase in the number of chondrocyte-like cells was observed at the margin of the intervertebral disc adjacent to the vertebral end plates, where the positive stained disc cells were accumulated in larger numbers (Figure 8).

**Figure 9:**
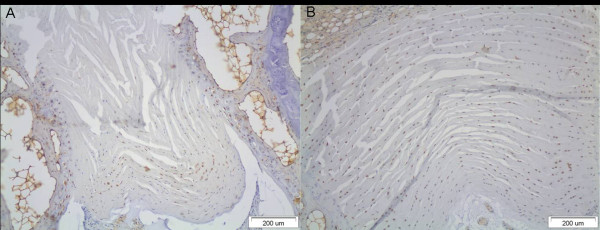
**Differences in the proportion of MMP-1 positive disc cells between A) the convex and B) the concave side of the deformity**.

In sections of group I, there were no marked differences in the proportion of MMP-1 positive cells between the convex and the concave side of the deformity (p = 0,6) (Figure 9). As the deformity progressed, the MMP-1 positive disc cells were slightly increased at the concave side, while they were significantly increased at the convex side, where the compression forces were higher. In group II, there was a statistically significant difference of the positive cells between the convex and the concave side (p < 0,01) (Figure 9). At the concave side of group III, there was a remarkable reduction in the number of disc's cells and a severe degradation of matrix microstructure (Table 2).

**Table 2:** Shows the mean score between the convex and concave side of the disc in the three examined groups and the statistical significance of the score difference.

	Group I	Group II	Group III
**Convex**	0,9	2,8	**3**
**Concave**	1,4	2,1	**1,2**

**p value**	**0,6**	**<0,01**	**<0,01**

Note the low score at the concave side of group III, where there was a remarkable reduction in the number of disc's cells and a severe degradation of matrix microstructure.

## Discussion

Intervertebral disc degeneration is characterized by a number of changes linked to the degradation of the extracellular matrix [20-22]. This loss of tissue integrity has been associated with an increase in the expression of MMPs of disc's cells. Indeed, a number of MMPs, such as MMP-1, -3, -13 has been reported to be expressed in aged and degenerated discs. Among them, MMP-1 seems to be crucial as it is expressed by the majority of the disc's cells [21] and can degrade several extracellular matrix components, such as, fibrillar collagens, gelatines, proteoglycans, fibronectin, laminin, etc. [23]. Roberts et al. by using immunohistochemical techniques have shown that MMP-1 is localized in the disc's cells [21]. MMP-1 was also found to be expressed in some cells in non-degenerated discs, indicating that it plays a role in normal tissue homeostasis, such in normal turnover of extracellulatr matrix components [24]. Its expression seems to be differentially regulated in humans in the various stages of development and during ageing. Weiler et al. have shown that MMP-1 is vaguely expressed in the discs of foetuses, infants and adolescents, while it is found in the discs of young adults, usually in tissue clefts and chondrocyte-like cells [25]. In older adults the expression of MMP-1 was also intense; however a reduced number of positive cells were found in people over 60 years of age. In addition, they have reported an increased expression in degenerated (and especially herniated) discs, and particularly in clustered cells. Similarly, a number of studies from other groups have also shown that MMP-1 is over-expressed in degenerated discs [24,26,27]. MMPs are secreted from the cells as proenzymes or zymogens that can be activated by proteolytic degradation. Plasmin, a broad-spectrum serine protease can activate pro-MMPs to fully active MMPs. Interestingly, in traumatized and degenerated porcine intervertebral discs MMP-1 over-expression was accompanied by increased expression of plasmin [27], indicating that cascades of enzymatic activations can lead to local tissue degradation. Furthermore, collagen fragments that accumulate in the degenerated intervertebral disc can enhance the expression of MMP-1 [28], probably forming a self perpetuating cycle affecting tissue homeostasis. The same mechanism has also been proposed for aged skin, where collagen fragments can provoke an oxidative stress leading to MMP-1 overproduction that can be inhibited by antioxidant compounds [29].

In our study, an over-expression of MMP-1 has been observed after mechanical deformation of intervertebral discs, by a process simulating the scoliotic conditions. Interestingly, the increase in MMP-1 expression presented in our study is observed mostly in fibroblastic, annulus fibrosus cells. This is in contrast to previous studies indicating that the main increase in MMP-1 expression in degenerated discs is observed in nucleus pulposus cells [21,26]. This controversy can be due to species differences (human vs. rat), or can probably indicate different mechanisms in scoliosis and in disc degeneration disease. A positive effect of mechanical forces on MMP-1 expression or secretion has been reported also in various other models, from periodontal tissues to osteoblastic cells [30-32]. The increase of MMP-1 expression observed in this study seems to be dependent on the intensity of deformation and on the site of the tissue affected, as convex areas are more affected compared to concave ones. Similarly, Crean et al reported increased expression of MMP-2 and MMP-9 in the convexity of the curve of scoliotic discs [4]. Previous studies have shown that the intensity of mechanical forces can regulate the expression and activity of MMP-1. As an example, porcine aortic valve cups have been subjected to cyclic stretch and has been found that a physiological stretching, i.e. 10%, there was no increase of MMP-1. However, pathological stretch of 15% causes an intense increase of MMP-1 expression, also 20% extensive over-stretch still upregulated MMP-1 but in a lesser amount in comparison to 15% stretch [33]. In the same direction, it has been recently shown that circumferential constrain of *in vitro *formed nucleus pulposus tissues can lead to a decrease gene expression of aggrecan and collagen II and an increase expression of MMP-1 and ADAMTS-5, that can play a role towards remodelling and degradation of the tissue [34]. On the other hand, the expression of other MMPs (i.e. (MMP-2 and MMP-9) seem to be decreased after exposure of disc cells to mechanical forces [35]. Regarding the role of MMPs family in idiopathic scoliosis, there are some contradictory findings in the literature about the role of gene variants of IL-6 and MMP-3 and whether MMP-3 and IL-6 promoter polymorphisms constitute important factors for the genetic predisposition to idiopathic scoliosis [36,37].

In this study we observed changes in the numbers and types of cells in the various areas of the discs after a long-term deformation. In particular, a reduction of the number of cells was observed in discs subjected to intense force. The mechanism leading to the reduction of the number of cells (i.e. apoptosis) needs to be further investigated with the use of specific markers. Analogous data have been reported in mouse tails subjected to static bending [38]. Interestingly, in the areas of reduced cellularity a severe degradation of matrix microstructure has also been found. A plausible explanation can be the leakage of proteases from dying cells, which can catabolise extracellular matrix components. In addition, we observed that at the margins of intervertebral discs adjacent to the vertebral end plates a reduction of fibroblast-like cells and an increased number of chondrocytes positive in MMP-1 expression. Similarly, Court et al. have reported that in bended mouse tails and specifically in the inner concave annulus a change from a fibroblastic to a chondrocytic phenotype [38]. The authors provide three alternative explanations for this phenomenon: first, a preferential fibroblast cell death; second, a chondrocytic migration in this area; and third, a differentiation of fibroblasts to chondrocytes. Obviously, these possibilities remain to be experimentally validated. The former hypothesis is partly supported by the increased apoptosis found in scoliotic discs [39]; however, differences in apoptotic rates between fibrocyte-like and chondrocyte-like cells need to be investigated.

Mechanical forces affect several aspects of tissue homeostasis by activating numerous intracellular signalling pathways. Static mechanical deformation can stimulate the proliferation and expression of transcription factors regulating the differentiation of periodontal ligament fibroblasts [40,41]. This is achieved by the activation of members of the MAPK family (ERK, JNK and p38) and the upregulation of the components of the AP-1 transcription factor, i.e. c-Fos and c-Jun [41,42]. On the other hand, interstitial flow stimulates the expression of MMP-1 in smooth muscle cells via ERK and JNK activation [43], while in monocytes MMP-1 induction is regulated by AP-1 [44]. However, in intervertebral disc cells MMP-1 can also be overexpressed by the pro-inflammatory cytokine TNF-α, via the p38 and JNK pathways [45,46]. Accordingly, the involvement of the above mentioned pathways in the regulation of MMP-1 in scoliotic disc needs further investigation in vitro and in vivo.

## Conclusion

The present study showed that an experimentally induced scoliotic deformity on a rat tail intervertebral disc results in over-expression of MMP-1, which is dependent on the degree of the deformity and follows a dissimilar distribution between the convex and the concave side. Furthermore, disc degeneration is associated with an over-expression of several MMPs, and their inhibitors (TIMPs), as their balance is decisive in the process of matrix degradation. The present study addressed only MMP-1. Our future studies will focus on the comparison of the expression of other MMPs and TIMPs, as well as other degradative molecules, such as aggrecanases.

## Abbreviations

MAPK: Mitogen-activated protein kinases; ERK: Extracellular signal-regulated kinases; JNK: c-Jun N-terminal kinases; AP-1: Activation protein-1; TIMPs: Tissue inhibitor of Metalloproteinases.

## Authors' contributions

TBG conceived the idea of the presented study, performed part of the experiment, contributed in the interpretation of data and in the drafting of the manuscript, obtained funding and gave the final approval. ESV performed part of the experiment, performed part of literature review, contributed in the interpretation of data and in manuscript drafting. AK contributed in the experiment and in the interpretation of data. LK performed the histological and immunohistochemical analysis and contributed in manuscript drafting. DK performed part of literature review, contributed in the interpretation of data and in manuscript drafting. All authors have read and approved the final manuscript.
